# Long-term prognosis of schizophrenia in a Danish context

**DOI:** 10.1192/j.eurpsy.2025.132

**Published:** 2025-08-26

**Authors:** H. G. Hansen, M. Starzer

**Affiliations:** 1 Department of Clinical Medicine, University of Copenhagen, Denmark, Copebhagen Research Center for Mental Health; 2 Department of Clinical Medicin, University of Copenhagen, Denmark, Copenhagen Research Center for Mental Health, Hellerup, Denmark

## Abstract

**Abstract:**

The Danish OPUS trial was originally a randomized controlled trial testing Early Intervention Services among individuals diagnosed with schizophrenia spectrum disorders between the years 1998-2002. Today, the OPUS trial includes a unique cohort of individuals (n=578) longitudinally followed and clinically assessed for more than two decades after a first diagnosis within the schizophrenia spectrum. Using longitudinal Danish register data and clinical data from the Danish OPUS cohort, we explored the effects of early intervention services including symptom remission, use of antipsychotics, hospitalization, global functioning and other aspects of life including social relations, parenthood, and mortality.

**Results:**

Investigating modern-era treatment facilities in 20 years of follow-up of the OPUS trial, we found no evidence for long-term disease-modifying effects from two years of Early Intervention Services compared to Treatment as Usual. While a majority of participants experienced a reduction and stabilization of psychotic symptoms over time it seems half of all the participants experienced no significant improvements in negative symptoms. Still, 17% were in clinical recovery, and 40% were in symptom remission. Combining clinical data from the Danish OPUS cohort with the Danish registers, we found that 29% (n=120) of individuals were in current treatment with antipsychotic medication after 20 years of follow-up. A total of 36% (n=51) of the clinical sample were in remission of psychotic symptoms and off antipsychotics with better clinical outcomes compared to individuals in non-remission off/on medication. Additionally, a substantial proportion of 38% (219 out of 578) of individuals in the OPUS cohort had become parents over two decades, and on average, they had better functional and clinical outcomes than their counterparts without children. A mortality rate of 14.2 was found. Suicide was the single most common cause of death, but death due to natural causes and death due to unnatural causes made of roughly half of all deaths each.

**Conclusions:**

While many patients may achieve remission of psychotic symptoms, our findings suggest negative symptoms are of a much more chronic nature. Investigating the use of antipsychotics over 20 years a substantial proportion were in remission and off medication. Since an observational study design cannot differentiate between cause and effect, these individuals still set a benchmark for good outcomes in schizophrenia. Also, 38% of individuals had become parents over two decades, and cross-sectionally, they had better illness prognosis and lifetime course than their counterparts without children. Finally, one in seven of the participants in the OPUS trial had died during follow-up. Suicide is a vital problem even many years after diagnosis and suicide-preventive measures are needed in the later course of illness.

**Disclosure of Interest:**

None Declared

